# Acid exposure induces adaptive resistance in *Klebsiella pneumoniae* to combination treatment with antibiotics and biofilm-disrupting surgical lavage

**DOI:** 10.1128/spectrum.00243-26

**Published:** 2026-07-16

**Authors:** Jordan L. Chirumbolo, Amanda Hutsell, Amar Kosovac, Isabella Ihemis, Melissa J. Whitford, Juliane Nguy, Rowan J. Wolf, Damir Sarajlic, Topher Hunter, Matthew F. Myntti, Terri N. Ellis

**Affiliations:** 1 Department of Biological Sciences, University of North Florida, Jacksonville, Florida, USA; 2 NextScience LLC, Jacksonville, Florida, USA; Houston Methodist Hospital, Houston, Texas, USA

**Keywords:** *Klebsiella*, porins, antibiotic resistance, antibiotic tolerance

## Abstract

Surgical irrigation solutions remove bacteria and disrupt biofilms to prevent nosocomial infections. This study evaluated the impact of combined treatment of a biofilm-disrupting lavage solution and antibiotic therapy. Bacteria treated with antibiotics and/or lavage were evaluated via broth microdilution and checkerboard assays. *Staphylococcus aureus* was strongly inhibited by all doses of surgical lavage, while combination treatments of *Escherichia coli* increased antibiotic susceptibility by more than 10-fold. However, combination treatments of *Klebsiella pneumoniae* resulted in specific dose regimens with diluted lavage solution that eliminated the bacteriostatic effect, such that significant bacterial growth occurred at 4× minimum inhibitory concentration treatments. This effect was observed across multiple strains of *K. pneumoniae* and multiple classes of antibiotics. Analysis of the individual components of the lavage solution revealed this to be a pH-related effect. *Klebsiella* grown in pH 4.5 media replicated the observed increase in minimum inhibitory concentration (MIC) without the addition of the other lavage components. This adaptive resistance response was correlated with activated stress responses, alterations in membrane polarization, and altered transcription of outer membrane porins. Transcription of *ompA* and *lpp*, which stabilize the outer membrane, was upregulated, while transcription of the alternative maltoporin *lamB* was halted. Deletion of the *iroA* siderophore also enhanced growth in bacteriostatic concentrations of antibiotics at low pH. Together, these data strongly suggest that *Klebsiella* has a distinct ability to mount a rapid adaptive response to an acidic environment via changes in membrane permeability, porin, and siderophore gene expression to enhance survival in the presence of both disinfecting agents and antibiotics.

**IMPORTANCE:**

Disinfecting washes that disrupt biofilms are commonly used in hospital settings to prevent the establishment of bacterial infection. This study investigated how combined exposure to a biofilm-disrupting surgical wash and antibiotics affected bacterial susceptibility to these bacteriostatic agents. While the response to *Staphylococcus aureus* and *Escherichia coli* was additive and fully bacteriostatic, we found specific combinations of antibiotic and diluted disinfecting agent that enhanced bacterial growth of *Klebsiella pneumoniae*, evidence of an adaptive resistance response. This response was found to be specific to *Klebsiella*, was initiated by a specific acidic pH range, and required altered transcription of major porins. These data indicate that *Klebsiella* can adapt to an acidic pH in ways that enhance resistance to both antibiotics and disinfectant agents. This adaptive response may be an important precursor to the evolution of fully resistant strains of *Klebsiella* via porin loss.

## INTRODUCTION

*Klebsiella pneumoniae* is a prominent cause of nosocomial bacterial infections, including pneumonia, urinary tract infections, and sepsis (1). As a key member of the ESKAPE pathogen group (2), *Klebsiella* exhibits a high rate of antibiotic resistance, including resistance to carbapenems, and has the potential to achieve pan-antibiotic resistance. *Klebsiella* exhibits resistance to antibiotics via several mechanisms, including the alteration of expression of outer membrane porins, which serve as non-specific facilitated diffusion channels allowing nutrients such as sugars, as well as beta-lactam antibiotics, to transit the bacterial outer membrane (3, 4).

Resistance to antibiotics is commonly thought to be solely the result of genetic mutations and acquired resistance genes. However, recent studies have highlighted the ability of bacteria to respond rapidly to antibiotic exposure through changes in gene expression that allow for survival or even growth at otherwise bacteriostatic antibiotic concentrations. Tolerant or persister cells may be able to survive these antibiotic exposures by slowing or entirely halting growth, while a transient response that allows for active bacterial growth in the presence of antibiotics is termed an adaptive resistance response (5, 6).

Porin loss clinical isolates of *Klebsiella* often do not express OmpK35 and/or OmpK36, the two porins that best allow for movement of beta-lactams across the membrane. These isolates are most commonly the result of mutations within the porin genes. However, porin expression is highly regulated and changes in the expression of these genes have also been associated with enhanced resistance (7). This active alteration of the permeability of the outer membrane via porins is particularly pronounced in *Klebsiella* and has been shown to enhance beta-lactamase-mediated ESBL- and KPC-based carbapenem resistance (7–9).

In addition to being highly antibiotic resistant, *Klebsiella* is highly effective at evading immune killing. This is mediated in large part via the polysaccharide capsule, which enhances serum resistance and limits phagocytosis. *Klebsiella* also produces multiple siderophores, allowing for the effective scavenging of critical iron and other metal micronutrients (10). Hypervirulent *Klebsiella* strains exhibit enhanced production of capsule and other evasion-focused virulence factors that allow for persistence and potential for the formation of biofilms that further limit penetration of antibiotics to the bacteria (1, 11).

In the hospital setting, there has been increased focus on treatments to prevent the colonization of wounds and surgical incisions with nosocomial pathogens. One approach is the use of biofilm-disrupting lavages applied topically at surgical sites. Treatment with these agents delivers a combination of chelators, detergents, and antibiotics to soft tissues. The intended effect is to disrupt and/or kill bacteria that might otherwise colonize, establish a biofilm, and progress to a nosocomial infection (12).

In this study, we investigated the combined effect of a biofilm-disrupting surgical lavage and antibiotic therapy on *K. pneumoniae*. We found that combination treatment of *K. pneumoniae* with both surgical lavage and cephalothin resulted in specific dose regimens with diluted lavage solution that resulted in an adaptive resistance phenotype, such that significant bacterial growth occurred at 4× minimum inhibitory concentration (MIC) treatments. This adaptive resistance was observed across multiple strains of *K. pneumoniae* and multiple antibiotics with differing cellular targets. Analysis of the individual components of the surgical lavage solution revealed this adaptive resistance to be driven by exposure to an acidic pH and to correlate with the activation of stress regulators, alteration to membrane polarization, and profound downregulation of the outer membrane porin *lamB*, without significant changes in expression of *ompK35* or *ompK36*. Deletion of the *iroA* siderophore also enhanced growth in bacteriostatic concentrations of antibiotics at low pH. The results of this study demonstrate the distinctive ability of *K. pneumoniae* to actively alter the outer membrane and outer membrane porin expression to enhance survival in the presence of both disinfecting lavage agents and antibiotics. Fundamental environmental conditions, such as pH, can alter the expression of porins and other virulence factors, allowing for an adaptive resistance response that may be a key first step in the evolution of resistant strains and the emergence of antibiotic-resistant porin-loss isolates.

## MATERIALS AND METHODS

### Bacterial strains, media, antibiotics, and biofilm-disrupting lavage

Bacterial strains used in this study are listed in Table 1. All cultures were grown from frozen stocks at 37°C with agitation (200 rpm) in Lysogeny Broth (Miller) (LB) (Difco, Detroit, MI). Deletion mutants were supplemented with maintenance antibiotics when appropriate. All antibiotics and chemicals are from Thermo Fisher Scientific (Haverhill, MA) unless otherwise noted. Xperience Advanced Surgical Irrigation solution (32.5 g/L citric acid, 31.3 g/L sodium citrate, and 1.0 g/L sodium lauryl sulfate in water) (13, 14), and all related formulations were provided by Next Science LLC (Jacksonville, FL). pH measurements of the media were made using a Mettler Toledo S400 Benchtop pH Meter (Columbus, OH). Bacterial growth in liquid cultures is measured at OD_600_ using an Eppendorf Biophotometer (Hamburg, Germany). Generation time was calculated from the specific growth rate obtained by fitting a straight line to a plot of the natural log of optical density versus time over the exponential phase of growth. The slope of this line (*µ*, in min^−^¹) was then used in the equation g=ln⁡(2)/μ to determine the doubling time in minutes.

**TABLE 1:** Strains used in this study

Bacterial strain	Description	Source
*E. coli* 31165	O139	ATCC
*E. coli* 43886	O29, heat-labile enterotoxin	ATCC
*E. coli* 43896	O78:H2, heat-labile enterotoxin	ATCC
*E. coli* 31616	O9 Diarrhetic *E. coli*	ATCC
*K. pneumoniae* 43816	Serotype O1:K2	ATCC
*K. pneumoniae* 2342	Bla-NDM, L-KPC	ATCC
*K. pneumoniae* 2524	Oxa48	ATCC
*K. pneumoniae* 2470	Bla NDM	ATCC
*K. pneumoniae* 43816 ΔwbbO	O-antigen knockout	(15)
*K. pneumoniae* 43816 ΔwcaJ	Capsule knockout	(16)
*K. pneumoniae* 43816 ΔybtS	Yesiniabactin knockout	(17)
*K. pneumoniae* 43816 ΔiroA	Salmochelin knockout	(17)
*K. pneumoniae* 43816 DiroA Δytbs	Yersiniabactin and Salmochelin knockout	(17)
*K. pneumoniae* 43816 ΔentB	Enterobactin and Salmochelin knockout	(17)
*K. pneumoniae* 43816 DentB Δytbs	Enterobactin, Yersiniabactin, and Salmochelin knockout	(17)

### Minimum inhibitory concentration assay

Broth microdilution assays were used to determine the MIC of bacteria exposed singly to an antibiotic (18). Briefly, 96-well plates were seeded with LB broth containing 10^3^ CFU/mL bacteria. Wells containing only LB and bacteria were used as controls for normal growth. Antibiotics were added in a 2-fold serial dilution, and the plate was incubated overnight at 37°C. Growth was measured at OD_600_ using a Biotek Gen5 plate reader (Winooski, VT). The lowest antibiotic concentration with no increase in optical density over a broth-only control was used as the MIC for each treatment.

For pre-treatment experiments, bacteria were initially grown overnight in the pretreatment broth (standard LB [Miller], LB [Miller] pH 4.5, or LB with 1:16 diluted Xperience). Bacteria were then pelleted (10,000 rpm for 10 min) and resuspended in a new media formulation, with a different pH or +/− Xperience to an appropriate OD_600_ and seeded into wells for a standard MIC assay. All dilutions for the MIC were done using the second media formulation.

### Membrane permeability and membrane potential assays

Integrity of the bacterial membrane was determined by a disc diffusion assay described by Llobet et al. (19). Sterile filter discs containing 80 μg of novobiocin were overlaid on lawns of bacteria on LB agar adjusted to either pH 7.0 or pH 4.5. Plates were incubated overnight at 37°C, and the diameter of the zone of inhibition was measured in millimeters.

Membrane depolarization was evaluated using a DiSC_3_(5)-fluorescence assay using the protocol from Buttress et al. (20). Briefly, log-phase cultures were adjusted to an OD_600_ of 0.5 and resuspended in the appropriate growth media supplemented with 0.5 mg/mL fatty-acid-free BSA (Sigma-Aldrich). Cells were seeded into black 96-well plates, and DiSC_3_(5) dissolved in DMSO was added to a final concentration of 0.5 μM. Fluorometric measurements were taken every 2 min, with shaking in-between readouts, using a BMG FLUOstar Omega multimode plate reader (Cary, NC) with filters for 610 nm excitation and 660 nm emission. Baseline membrane potential was measured for 20 min before adding 10 μg/mL colistin. Fluorometric measurements were then observed for an additional 20 min. Readings were normalized by subtracting average values for samples containing only bacteria and 10 μM DiSC_3_(5) diluted with the corresponding LB with fatty-acid-free BSA. *N* = 6 per treatment.

### Checkerboard assay for antimicrobial interactions

Checkerboard assays were performed to evaluate the combined effect of two antimicrobial treatments. Checkerboard assays were performed as described by Bellio et al. (21). Briefly, concentrated stock solutions were made of the antibiotic- and biofilm-disrupting solution in LB media. These solutions are serially diluted 2-fold either across the rows or down the columns of the plate, such that 77 distinct combinations of concentrations of the two agents are established. All wells are then seeded with broth containing 10^3^ CFU/mL bacteria. Each plate incorporates control wells with either bacteria alone or bacteria with each concentration of one agent. A mirror plate containing all dilutions without bacteria was used as a no-growth control and to determine background optical density. Plates are incubated overnight at 37°C, and growth was measured at OD_600_ using a Biotek Gen5 plate reader (Winooski, VT, USA).

### Whole genome sequencing and qPCR of gene transcripts

Bacteria were grown in LB (Miller), LB (Miller) pH 4.5, or LB (Miller) with 1:16 Xperience. Bacteria were either grown for 24 h and then sampled, or for 5 days with subculture into fresh media every 24 h. Cells were pelleted, preserved in Zymo DNA/RNA Shield (Irvine, CA), and sent for DNA extraction and whole genome sequencing by Plasmidsaurus (Louisville, KY). WGS was conducted using a hybrid approach using both Oxford Nanopore (long-reads) and Illumina (short-reads) platforms. Genomes were compared against the published reference sequence GCF_016071735.1 for *K. pneumoniae* 43816 (22). Details of the bioinformatics analysis, genome assembly, and variant-calling pipelines can be found on the Plasmidsaurus website (23). Unique SNPs were identified from each variant list by comparison with genome results for cultures grown in neutral LB without antibiotics.

For qPCR, RNA was extracted from cultures grown to an OD_600_ of 1.0 by treatment with RNA Protect, followed by extraction using the RNeasy + kit (Qiagen). Complementary DNA (cDNA) was reverse transcribed from 1 μg of total RNA using random hexamer primers and the Protoscript II reverse transcriptase kit (New England Biolabs). Quantitative polymerase chain reaction (qPCR) was performed using Luna qPCR Master Mix (New England Biolabs) on an Eppendorf Mastercycler Realplex 2 (Hamburg, Germany). Data were analyzed using the ΔΔCT method (24). Gene expression was normalized to *gapA* and the untreated growth strain. Primers used are provided in Table S1.

### Statistical analysis

All experiments were performed with *n* > 3. Statistical significance was determined using a one-way ANOVA and Tukey’s post hoc test using the Real Statistics Resource Pack software (Release 9.4.5) for Microsoft Excel (25). For all figures *P < 0.05, **P < 0.005, ***P < 0.0001. Statistical comparisons of transcriptional changes were conducted using ΔCt values to allow for comparison relative to the expression of each gene in untreated (LB pH 7) culture.

## RESULTS

### The bacteriostatic effect of combined antibiotic treatment and a biofilm disruptor is bacterial species-specific

**Fig 1:**
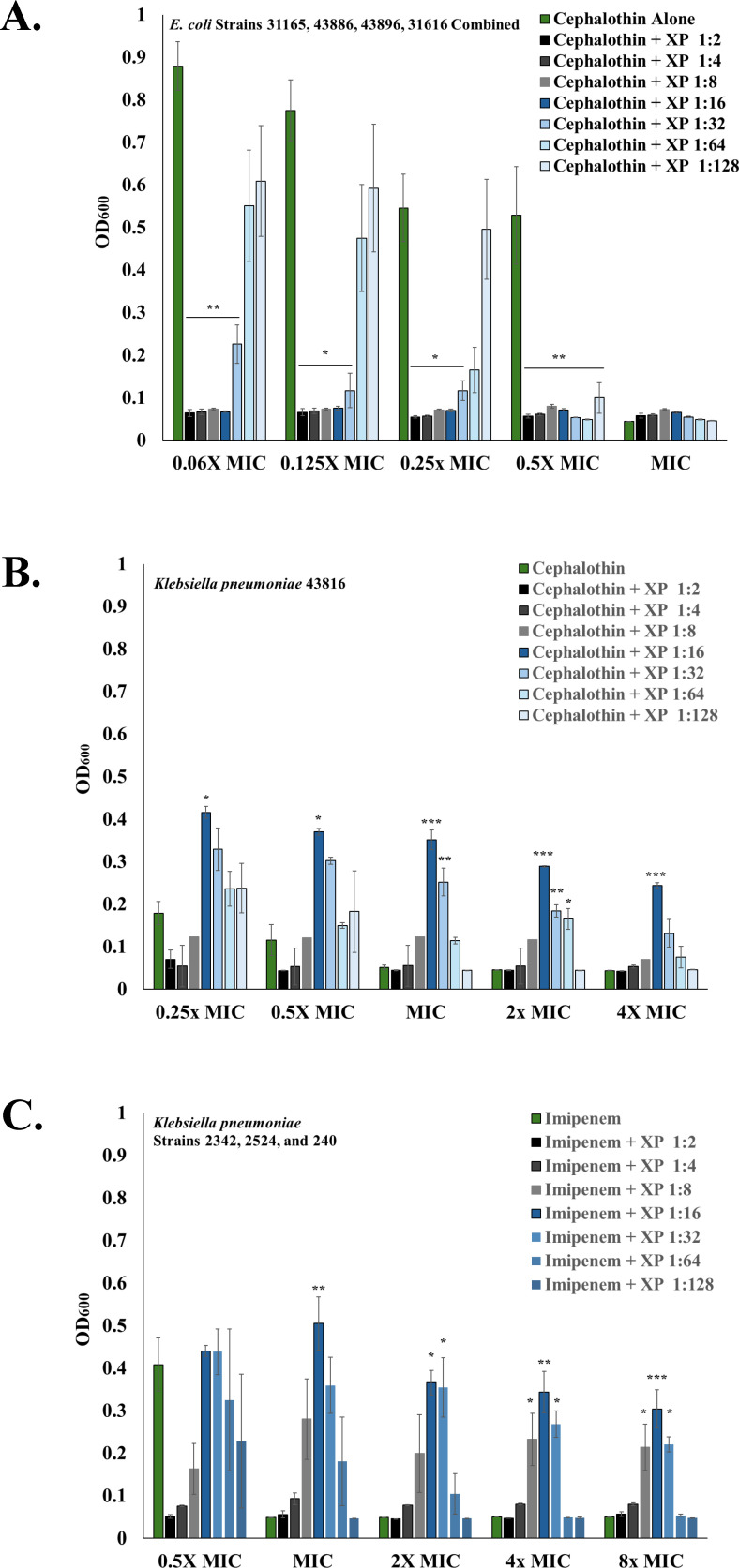
Survival and growth in co-treatment with antibiotics and Xperience is bacterial species-specific. Checkerboard assays were used to evaluate the effect of co-treatment with cephalothin and Xperience on (**A**) *E. coli* strains 31165, 43886, 43895, and 31616, (**B**) *K. pneumoniae* 43816, (**C**) Imipenem and Xperience on *K. pneumoniae* strains 2342, 2542, and 2470. Figures A and C represent the average across all strains. *N* = 3 independent assays. Bars represent the standard error from the mean. Statistical comparisons are against antibiotic-only treatment. *P < 0.05, **P < 0.005, ***P < 0.0001.

Checkerboard assays were used to determine the combined effect of the antibiotic cephalothin with the biofilm-disrupting surgical lavage Xperience. Tests of multiple strains of *E. coli* (listed in Table 1) revealed a consistent additive effect across bacterial strains. Figure 1A shows the combined results across four different *E. coli* strains. The biofilm disruptor Xperience enhanced the bacteriostatic effect of sub-MIC concentrations of antibiotic. Doses of cephalothin of less than one-tenth the MIC concentration prevented *E. coli* growth when combined with equivalent diluted concentrations of Xperience. Tests of *S. aureus* demonstrated a bacteriostatic effect in response to all concentrations of Xperience, including a 1:128 dilution of Xperience without added antibiotic (Fig. S1). These data demonstrate the bacteriostatic and additive potential of antibiotics and biofilm disruptors together.

A significantly different trend emerged when the combined treatment was tested against *K. pneumoniae* 43816 (Fig. 1B). When exposed to minimally diluted (up to 1:4) Xperience, an additive bacteriostatic effect was seen with sub-MIC antibiotics, like that seen with *E. coli*. However, greater dilutions resulted in significant growth of the bacteria, even at above the antibiotic MIC concentrations. In particular, the combination of a 1:16 dilution of Xperience and up to 4× MIC resulted in significant bacterial growth. While FIC calculations did not reach the formal threshold for an antagonistic effect (data not shown), the active growth of the bacteria in combinations that are bacteriostatic for other bacterial species is highly concerning.

To determine if this was a strain-specific phenomenon, we tested three other strains of *K. pneumoniae* (listed in Table 1). These strains have acquired a variety of beta-lactamase resistance genes and hence were tested against imipenem, a beta-lactam to which they are susceptible. Figure 1C shows the combined results across these strains. These data demonstrate the same trend as seen in Fig. 1B, with all strains exhibiting significant growth when exposed to the same range of Xperience dilutions, with peak growth occurring in the 1:16 dilution and up to 8× imipenem MIC concentrations. This indicates that the ability of *Klebsiella* to tolerate and actively grow in what should be bacteriostatic concentrations of antibiotic when added to the diluted lavage solution is species-specific rather than strain-specific.

**Fig 2:**
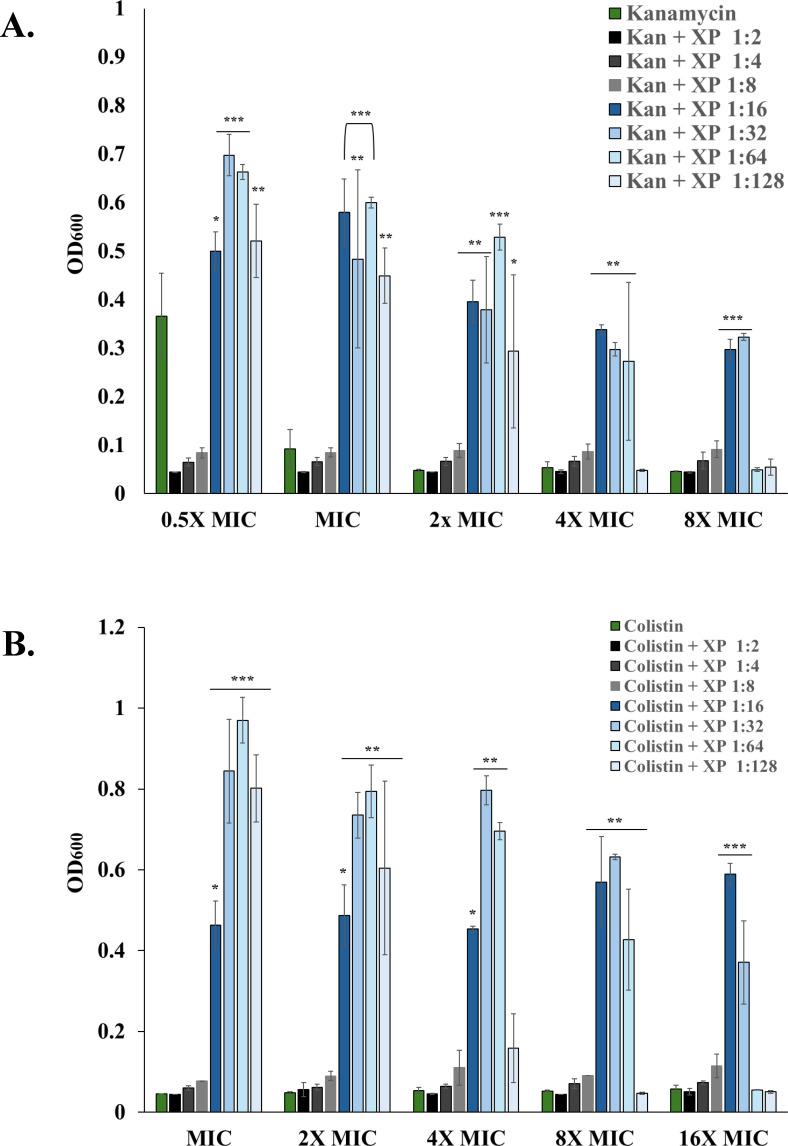
Exposure to diluted Xperience allows for growth in bacteriostatic concentrations of multiple classes of antibiotics. Checkerboard assays were used to evaluate the effect of co-treatment of *K. pneumoniae* 43816 with Xperience and (**A**) kanamycin and (**B**) colistin. *N* = 3 independent assays. Bars represent the standard error from the mean. Statistical comparisons are against antibiotic treatment alone at the same dose. *P < 0.05, **P < 0.005, ***P < 0.0001.

We then investigated if this response in *Klebsiella* was limited to only beta-lactam antibiotics. Checkerboard assays were performed with *K. pneumoniae* 43816 with either kanamycin (Fig. 2A), a ribosome inhibitor, or colistin (Fig. 2B), which disrupts outer membrane LPS. Consistent with our previous experiments, significant growth of the bacteria was seen when exposed to Xperience diluted more than 1:8 in combination with up to 8× MIC concentrations of either antibiotic. Together, these data indicate that *K. pneumoniae*, unlike either *E. coli* or *S. aureus*, responds to the diluted biofilm disruptor in a way that allows for bacterial tolerance and active growth in the presence of otherwise bacteriostatic antibiotic concentrations. This effect is not dependent on either the drug mechanism or the site of action within the bacterial cell. Growth was observed in the presence of colistin, which targets the outer membrane directly, with beta-lactams that require transit across the outer membrane, and kanamycin, which must cross both membranes to reach the molecular target of action.

### The *Klebsiella*-adaptive resistance response to Xperience corresponds to a limited pH range

**Fig 3:**
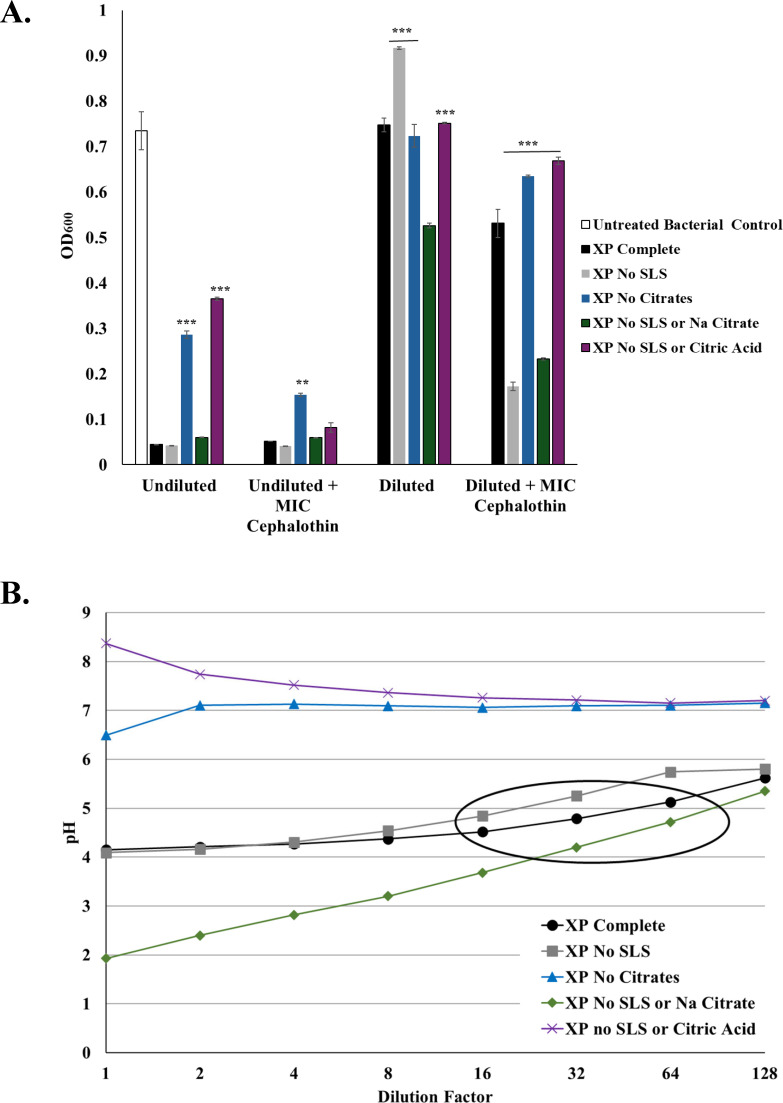
Treatment with chemical components of Xperience reveals that a specific pH range allows for *K. pneumoniae* growth. *K. pneumoniae* 43816 was incubated with Xperience formulations lacking specific ingredients in the presence and absence of the MIC concentration of cephalothin. (**A**) Bacterial growth was determined after 24-h incubation. *N* = 3 independent assays. Bars represent the standard error from the mean. Statistical comparisons are against the relevant treatment with complete Xperience in each group. *P < 0.05, **P < 0.005, ***P < 0.0001. (**B**) The pH of the different formulations of Xperience changes with dilution. Dilutions within the highlighted circle resulted in significant bacterial growth in the presence of MIC concentrations of cephalothin.

The biofilm disruptor Xperience is formulated from a combination of sodium lauryl sulfate (SLS), sodium citrate, and citric acid. We hypothesized that these components may not contribute equally to the *Klebsiella*-adaptive resistance response. To investigate the role of each component, Xperience formulations were created with subsets of the three ingredients. These solutions were then tested with *Klebsiella* and cephalothin. Figure 3A shows the results of exposing *Klebsiella* to these formulations in the presence and absence of cephalothin. When exposed to undiluted formulations of Xperience, removal of the SLS detergent did not alter the bacteriostatic effect. However, removal of all buffering citrates allowed for significant growth in both the presence and absence of MIC antibiotics. Significant growth was also observed in the formulation lacking both SLS and citric acid. However, this treatment remained responsive to MIC concentrations of antibiotics, indicating that the buffering effect of the citrates, rather than the effect of the detergent, is critical to the observed bacterial growth.

When exposed to the 1:16 diluted formulations of Xperience without antibiotics, growth to an OD of over 0.5 is seen in all treatments. The only diluted treatment with reduced growth compared to the untreated control had citric acid (SLS and Na Citrate are removed) added to the media, and a resulting pH below 4.0. Therefore, we investigated the change in pH of the growth media with the addition of each formulation. As seen in Fig. 3B, formulations that allow for active bacterial growth (within the circled range) correlate with a pH in the media of pH 4.2–5.0. The diluted citric acid treatment falls outside of this range, with a pH below 4.0, which may explain the lower optical density observed. The formulations lacking citric acid entirely retained a neutral pH.

When combined with cephalothin, the diluted Xperience formulations reveal the relative contribution of each component to the adaptive resistance response. Treatments that retained citric acid exhibited significantly lower bacterial growth, while formulations that removed the citric acid significantly enhanced growth over that of complete Xperience. Of note, reduced growth in the presence of antibiotics is seen in media with either a neutral pH or a pH below 4.2. The formulations that enhanced growth all had LB media with pH in the range of 4.2–5.0, as circled in Fig. 3B. These data indicate that the pH of the treatment solution may be a key trigger of the adaptive resistance response. Combination treatments outside this defined pH range allow for the bacteriostatic action of either the biofilm disruptor or the antibiotic.

### Acidic pH alters *Klebsiella* physiology and MICs

**Fig 4:**
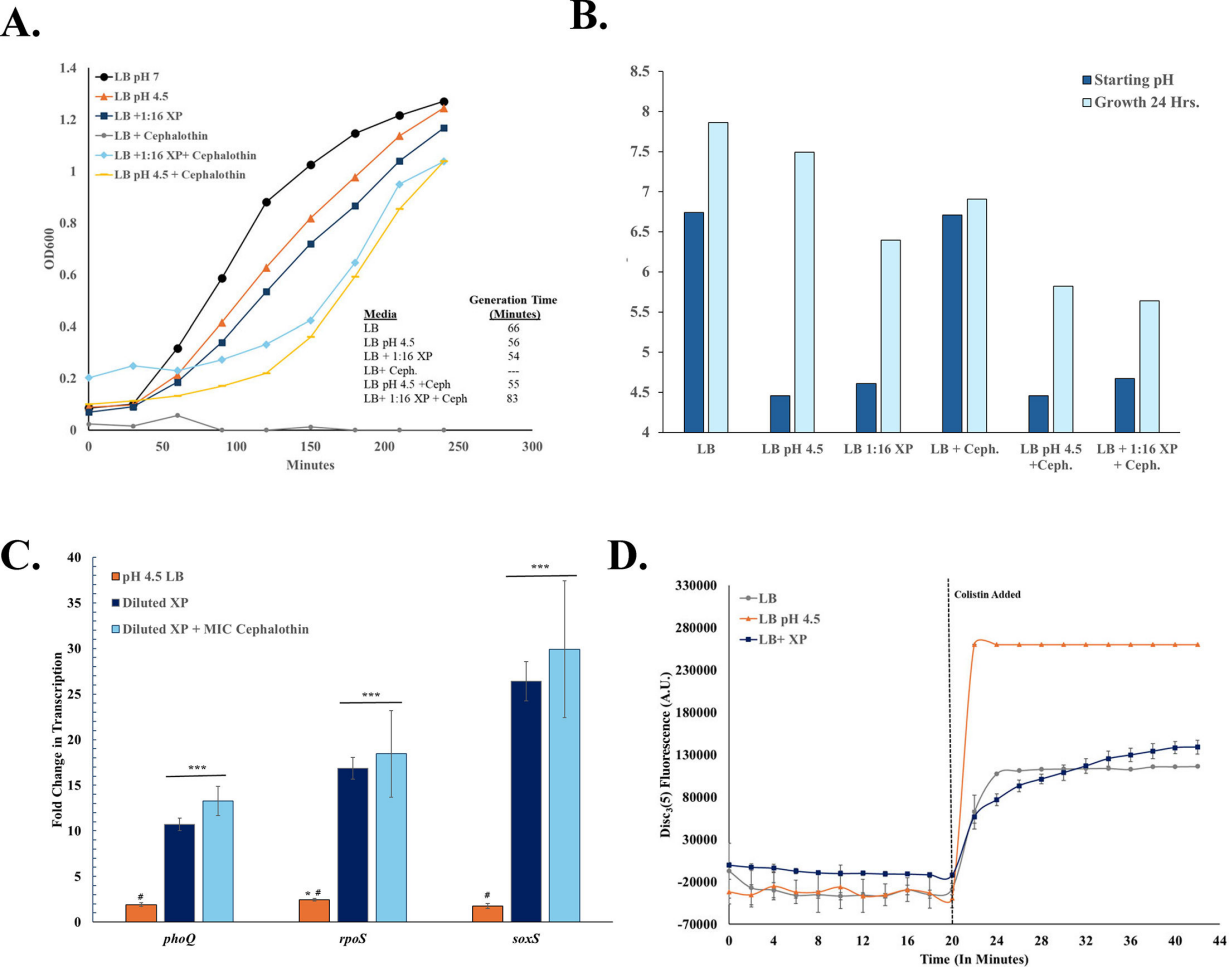
Acidic pH and XP induce a transcriptionally regulated adaptive resistance phenotype with a distinct XP-dependent membrane polarization profile. *K. pneumoniae* 4816 was grown in neutral LB, pH 4.5 LB, or 1:16 XP with or without 16 μg/mL cephalothin. (**A**) OD_600_ of cultures was determined 30 min over the course of 4 h. Generation time was calculated from the specific growth rate of the exponential portion of each curve. (**B**) pH of cultures at the start and end of 24 h incubation in each growth medium. (**C**) Transcription of three stress response regulators was determined by qPCR. Relative transcriptional levels were determined using the ΔΔCt method, comparing against the housekeeping gene *gapA* and the untreated growth strain in neutral LB. *N* = 3 independent assays. Bars represent standard error. Statistical comparisons were conducted using ΔCt values to allow for comparison relative to the expression of each gene in untreated culture. *P < 0.05, ***P < 0.0001 relative to neutral LB. # *P* < 0.05 as compared to cultures containing dilute XP. (**D**) *K. pneumoniae* equilibrated with the voltage-sensitive dye DiSC_3_(5) for 20 min and exposed to 10× MIC colistin (dashed vertical line). Depolarization is measured via an increase in fluorescence measured every 2 min. Flat readings after colistin addition represent maximum depolarization. *N* = 6 per treatment. Error bars represent standard deviation.

To determine whether the observed adaptive resistance response is a direct consequence of acidic growth conditions, we compared *K. pneumoniae* grown in diluted XP with bacteria cultured in LB medium adjusted from pH 7.0 to 4.5 using HCl. Growth curves, measured by OD, were then determined in the presence or absence of MIC levels of cephalothin. As shown in Fig. 4A, the adaptive resistance response of active growth in the presence of MIC concentrations of cephalothin was replicated in pH 4.5 LB media. Beyond this, three distinct growth patterns emerged. Cultures grown in either acidic LB or dilute XP exhibited an extended lag phase compared to neutral pH LB media. Although this lag phase was prolonged, once cultures entered the exponential phase, the generation time decreased below that observed in neutral pH LB. Addition of cephalothin at MIC further prolonged this lag phase. Notably, only cultures treated with both XP and cephalothin exhibited an overall lengthening of the exponential-phase generation time. These findings indicate that *K. pneumoniae* actively alters cellular processes during this lag phase in a manner distinct from each growth condition.

Because bacterial metabolic activity can alter media pH, we next measured pH changes during growth. As shown in Fig. 4B, *K*. *pneumoniae* increased the pH of all media in which it could survive over time. The greatest pH increase occurred in cultures initiated at pH 4.5, likely reflecting the lower buffering capacity of LB compared to the citrate-containing XP solution. In contrast, cultures grown in the presence of cephalothin exhibited a much smaller pH increase, even when reaching optical densities above 0.4. This suggests that the combination of acidic pH and antibiotic exposure induces an adaptive response that extends beyond the production of alkaline metabolic byproducts.

Acidic pH is known to activate global regulators and stress responses that may indirectly alter antibiotic susceptibility. To investigate which stress response pathways may be involved in this adaptive resistance, we evaluated transcription of three key regulatory genes that participate in multiple overlapping stress responses. Relative transcriptional levels were compared against the housekeeping gene *gapA* and the target gene as expressed in strain 43816 when grown in pH 7.0 LB media alone. Statistical comparisons were conducted using ΔCt values to allow for comparison relative to the expression of each gene in untreated (LB pH 7.0) culture.

As seen in Fig. 4C, treatment with dilute Xperience dramatically increased expression of the general stress sigma factor *rpoS*, the oxidative stress regulator *soxS*, and *phoQ*, the sensor of periplasmic and envelope damage. Notably, growth in pH 4.5 LB alone produced only a modest, although statistically significant, increase in *rpoS* expression compared to neutral LB. Addition of cephalothin to the dilute Xperience treatment did not further alter expression of these stress genes beyond the strong upregulation induced by dilute Xperience alone. These data demonstrate the breadth of transcriptional activation triggered by Xperience and suggest that these stress responses may be critically involved in the resulting adaptive resistance to cephalothin.

We next assessed the effect of acidic pH on antibiotic susceptibility across multiple drug classes. As shown in Table 2, the MICs of cephalothin, kanamycin, and colistin all increased 4-fold to 8-fold under acidic conditions, indicating that this pH-dependent effect broadly reduces the efficacy of multiple classes of antibiotics with different cellular targets. To determine whether acidic pH alters membrane permeability or efflux activity, we performed a novobiocin disc diffusion assay. Novobiocin susceptibility is a known indicator of RND-type efflux pump activity (26), and no significant differences were observed in neutral and acidic media (Table 2), suggesting that adaptive growth at low pH does not involve major changes in RND efflux pump function.

**TABLE 2:** Effect of acidic pH on antibiotic susceptibility of *K. pneumoniae* 43816

			Media resuspension assays			
**Antibiotic**	**LB pH 7.0**	**LB pH 4.5**	**LB pH 7→LB pH 4.5**	**LB pH 4.5→LB pH7**	**LB pH 7→XP**	**XP→LB pH 7**
Cephalothin	16	64	62.5	8	62.5	4
Kanamycin	16	64	MIC in μg/mL			
Colistin	1	8				
Novobiocin	145 (+13)	166 (+15)	Zone of Inhibition in mm (mean + SD)			

Because antibiotic uptake can be altered by changes in membrane polarization and proton motive force (27, 28), we next assessed membrane dynamics using the voltage-sensitive dye DiSC_3_(5). This lipophilic, cationic dye accumulates in polarized membranes and is released upon depolarization, resulting in increased fluorescence. Bacteria grown in each medium were equilibrated for 20 min with dye before the addition of 10× MIC colistin. As shown in Fig. 4C, cultures grown in neutral LB or pH 4.5 LB exhibited rapid and complete depolarization, reaching maximal fluorescence shortly after antibiotic exposure. Differences in maximal signal likely reflect pH-dependent baseline fluorescence effects. In contrast, cells grown in 1:16 XP showed a gradual, incomplete increase in fluorescence over the 20-min assay, revealing a unique XP-dependent membrane phenotype in which altered polarization may contribute to adaptive resistance by altering overall membrane permeability and limiting antibiotic transit. Consistent with this, differences in membrane polarization between XP-treated and control cultures were also observed across a range of 1×–10× MIC polymyxin concentrations (data not shown).

These observations suggest that the adaptive resistance phenotype is driven primarily by changes in gene expression rather than stable genetic mutations. To test this, we performed pretreatment experiments in which cells were grown overnight in one condition, then pelleted and transferred to a different medium prior to MIC testing. As shown in Table 2, cells pretreated in either acidic LB or dilute XP did not retain elevated resistance when transferred to neutral LB. In contrast, exposure to acidic pH or XP during antibiotic treatment induced a 4-fold to 8-fold increase in cephalothin MIC. This transient, exposure-dependent effect indicates that the adaptive resistance phenotype is reversible and tightly coupled to transcriptional responses triggered by the culture medium and broader environment.

To further assess whether prolonged exposure induces stable genetic changes, we performed whole-genome sequencing of cultures grown for 24 h or 5 days under each condition. As shown in Table S2, across all culture conditions and time points, the whole-genome sequencing data show remarkably little genetic divergence as compared to untreated cultures grown in parallel. A single upstream SNP at genome position 5215 was present in every treatment at both 1 and 5 days. Only a handful of additional variants were uniquely associated with individual conditions, and most of these are annotated as upstream or synonymous changes with modifier or low predicted impact, indicating minimal disruption of coding sequences. Even under combined low-pH and cephalothin exposure, where a small number of unique SNPs appear, these changes are rare events and do not suggest widespread selection for resistant mutants. Overall, the data support the conclusion that the bacterial populations remain genomically stable over 5 days of exposure to either acidic pH or dilute XP. Together, these results indicate that the adaptive resistance response is regulated at the level of gene expression, enabling a rapid and flexible response that dissipates once the acidic stimuli are removed, even in the presence of antibiotics.

### Major virulence factors do not contribute to the adaptive resistance of *Klebsiella* to antibiotics

**Fig 5:**
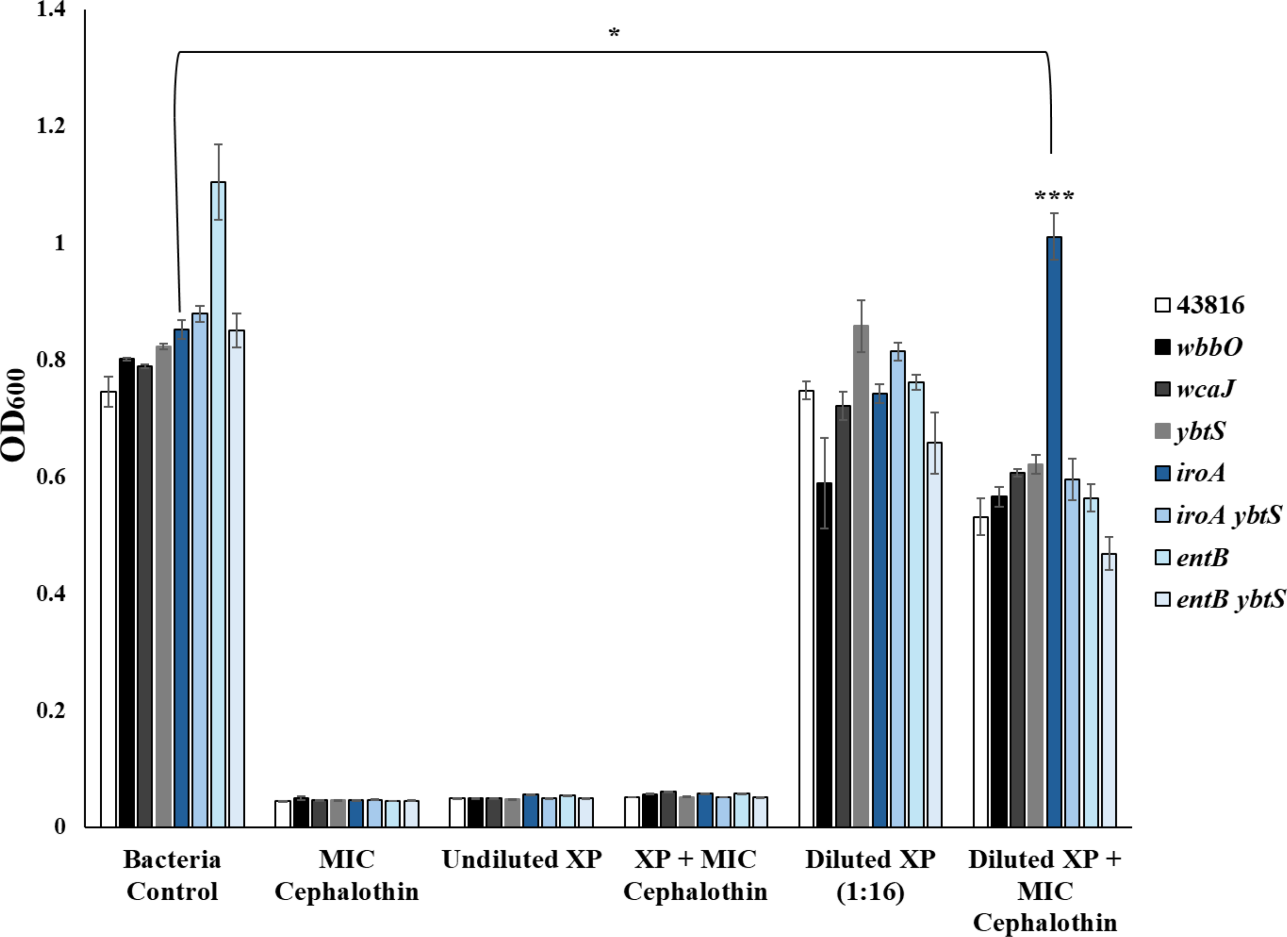
The contribution of major virulence factors of *K. pneumoniae* to survival in co-treatment. Isogenic knockouts of *K. pneumoniae* 43816 for LPS O-antigen (*wbbO*), capsule synthesis (*wcaJ*), and siderophores (*ybtS, iroA,* and *entB*) were tested in checkerboard assays combining Cephalothin and Xperience treatment. *N* = 3 independent assays. Bars represent the standard error from the mean. Statistical comparisons are against the bacterial growth control of each strain as well as between strains with the same chemical treatments. *P < 0.05, **P < 0.005, ***P < 0.0001.

*K. pneumoniae* contains several virulence factors that may play a role in the ability of antibiotics to access key cellular targets. To investigate the role of these virulence factors, we utilized isogenic knockouts in strain 43816 of the genes for LPS O-antigen synthesis (*wbbO*), capsule synthesis (*wcaJ*), and siderophores (*iroA, ybtS,* and *entB*) (Table 1). We hypothesized that loss of the LPS or capsule may increase the ability of the antibiotic to access the outer membrane of the bacterial cell. Alternatively, the siderophores may compete with the metal chelating agents present in Xperience, such that a lack of one or more may limit the ability of the bacteria to survive in the combined treatment. Each of these strains was tested in a full checkerboard assay using cephalothin as the target antibiotic. Figure 5 shows the combined results with either undiluted or 1:16 diluted Xperience.

Only one knockout, in *iroA*, caused a significant change in the pattern of growth. *iroA* allows for the secretion of salmochellin, the glucosylated form of the enterobactin siderophore encoded by *entB* (29). Contrary to our prediction, the *iroA* knockout exhibited significantly enhanced growth in the presence of diluted Xperience and cephalothin as compared to either the *iroA* untreated growth control or any other knockouts grown in the same combination treatment. Notably, neither the *entB* knockout, which is deficient in both enterobactin and salmochellin, nor the triple siderophore knockout (*entB ybtS*) showed any alteration in tolerance and growth as compared with the wild-type bacteria. Knockouts affecting either LPS O-antigen or capsule synthesis exhibited no change in the growth profile. These data demonstrate that neither capsule, LPS O-antigen, nor siderophore synthesis alone plays a significant role in this adaptive resistance response. Siderophore modification and the relative expression of specific forms of these molecules may be associated with the bacterial response to these treatments.

### *Klebsiella* porin expression is dramatically altered in conditions that trigger adaptive resistance to antibiotics

Antibiotic-resistant clinical isolates of *K. pneumoniae* are well-documented to exhibit the loss of porin channel proteins that allow for the transit of antibiotics across the bacterial outer membrane. Porin loss is generally regarded as a mechanism to enhance resistance via an acquired beta-lactamase gene, rather than as a standalone resistance mechanism (30). We investigated whether alterations in porin expression may be part of the *Klebsiella*-adaptive resistance response. Expression of six porin genes was evaluated by qPCR. As performed for the analysis of stress response genes, relative transcriptional levels were compared to *gapA* and the target gene in strain 43816 in pH 7.0 LB; statistical comparisons were conducted using ΔCt values to compare the relative expression of each gene in untreated culture.

**Fig 6:**
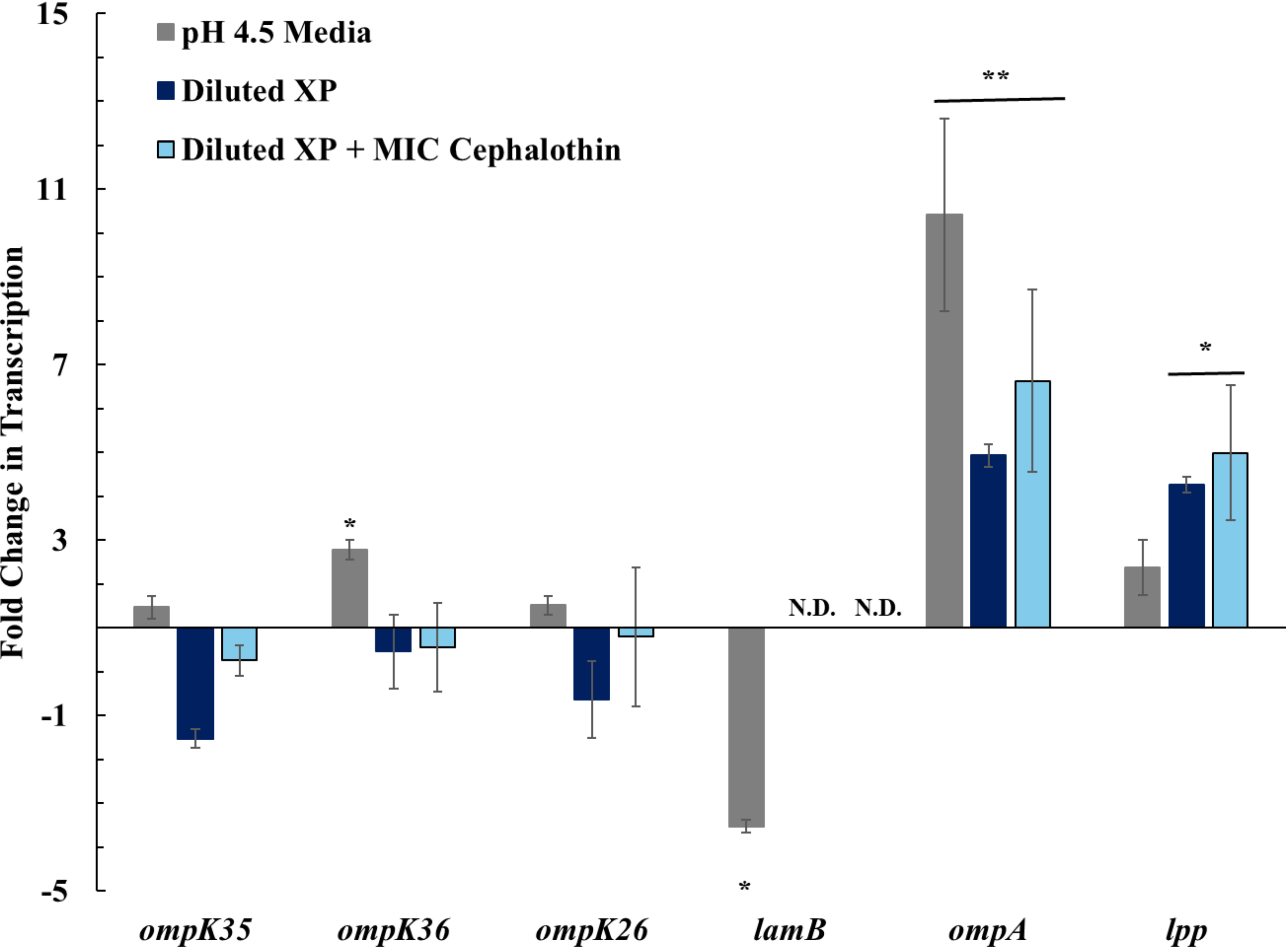
Transcription of outer membrane porins is altered by low pH and Xperience treatment. Transcription of six outer membrane porins was determined by qPCR. Relative transcriptional levels were determined using the ΔΔCt method comparing against the housekeeping gene *gapA* and the untreated growth strain. *N* = 3 independent assays. Bars represent the standard error from the mean. Statistical comparisons were conducted using ΔCt values to allow for comparison relative to the expression of each gene in untreated (neutral LB) culture. *P < 0.05, **P < 0.005, ***P < 0.0001. N.D. indicated not detected above thresholds for the assay.

Deletions of *ompK35* and *ompK36* are commonly seen in *Klebsiella* strains with porin loss-mediated resistance (31–34). However, no significant changes in expression were seen in either gene product when exposed to diluted Xperience (Fig. 6). Growth in acidic media alone did significantly upregulate *ompK36*, indicating that expression of these porins is impacted by an acidic pH but may be further regulated by other components of the lavage or antibiotic. No significant change was seen in the expression of *ompK26*, an alternative porin that has been shown to be significantly upregulated in porin loss isolates lacking either *ompK35* and/or *ompK36*. These data indicate that the porins commonly associated with porin loss-enhanced resistance may not be a significant aspect of the observed adaptive response in the presence of diluted Xperience.

Changes in the expression of *lamB*, *lpp,* and *ompA* have been previously demonstrated to compensate for the loss of permeability seen in porin loss-resistant strains (35). Our data demonstrate that these alternative porins exhibited significant changes in expression. *ompA,* which is known to stabilize the outer membrane, was significantly upregulated in response to all three treatments and demonstrated the highest upregulation in response to acidic pH. Expression of *lpp* was upregulated but did not reach the threshold of statistical significance when grown in acidic LB. However, *lpp* did exhibit significant upregulation when exposed to diluted Xperience, both with and without MIC antibiotics. *lpp* encodes for Braun’s lipoprotein, which is a key structural tether between the outer membrane and the peptidoglycan, further stabilizing the outer membrane (36).

Most significant is the extreme downregulation of *lamB* in all three conditions. The *lamB* gene in *Klebsiella* encodes the maltoporin LamB, an outer membrane channel that mediates uptake of maltose/maltodextrins and serves as an entry and control point for several antibiotics (37, 38). Expression of *lamB* is significantly downregulated at acidic pH alone and extremely downregulated in the presence of diluted Xperience. When exposed to diluted Xperience, *lamB* transcripts are so low that they are below the level of detection of the qPCR assay, indicating dramatic alterations in the expression of this porin. This pattern of upregulation of membrane-stabilizing porins and downregulation of porins that permit molecular transit across the membrane indicates that low pH and detergent exposure together likely activate distinct gene expression pathways that alter membrane stability and protein composition, enabling permissive growth of *Klebsiella* in otherwise bacteriostatic conditions and leading to an adaptive resistance response.

## DISCUSSION

The goal of this study was to evaluate the impact of combined treatment of a biofilm-disrupting lavage solution and antibiotic therapy on *K. pneumoniae*. Our hypothesis was that this combination would have an additive or synergistic bacteriostatic effect, like that seen in *E. coli* or *S. aureus*. While full-strength combination therapy was bacteriostatic, combination treatment with diluted lavage solution exhibited significant bacterial growth at antibiotic concentrations well above the MIC. This effect was observed across multiple strains of *K. pneumoniae* and multiple antibiotics, including drugs with cytoplasmic targets of action. Analysis of the individual components of the surgical lavage revealed this enhanced resistance to be a pH-related effect. *K. pneumoniae* grown in acidic LB exhibited elevated MICs to multiple antibiotic classes. This adaptive resistance response was correlated with significant changes in membrane polarization, upregulation of stress responses, and altered transcription of outer membrane porins. Transcription of *ompA* and *lpp*, which stabilize the outer membrane was upregulated, while expression of the alternative maltoporin *lamB* was halted. Deletion of the *iroA* siderophore also enhanced the ability of *Klebsiella* to actively grow in the presence of bacteriostatic concentrations of antibiotics in an acidic environment. Together, these data strongly suggest that *Klebsiella* can respond to an acidic environment via rapid transcriptional changes that alter porin gene expression and the overall membrane to allow the bacteria to survive exposure to multiple damaging agents.

Active adaptation and changes to virulence factor expression in an acidic pH may be an underappreciated theme of *Klebsiella* infections. *K. pneumoniae* actively colonizes multiple acidic body sites, including the gastrointestinal tract, vagina, and urinary tract (39), which are environments where pH-responsive regulatory systems like PhoPQ and RpoS would be activated. Lowered pH of interstitial fluids is a known complication of diabetes, and patients with diabetes are at increased risk for *Klebsiella* pyogenic liver abscess and soft tissue infections (40), suggesting that acidic tissue microenvironments may facilitate infection. Supporting the importance of acid tolerance for colonization, clinical suppression of gastric acid by proton pump inhibitors has been shown to enhance *Klebsiella* colonization of the GI tract (41), demonstrating that gastric acidity normally serves as a barrier to colonization. A survey by Koskinen et al. (42) of carbapenem-resistant nosocomial isolates of *Klebsiella* documented bacterial survival in environments with a pH as low as 4.0, which is within the pH range observed here to prompt an adaptive resistance response. In *E. coli,* the acid-tolerance response activates stress regulators, including *rpoS* and *phoQ*, and is required for survival in the mouse intestine (43). Our data further indicate that the stress response is not to pH alone but can modulate the activation of different response pathways based on ionic strength and chemical composition of the overall environment. Together, these studies demonstrate that the acid tolerance response of *Klebsiella* is a potent contributor to both host colonization and adaptive antibiotic resistance, with acid-responsive regulatory systems enabling the bacterium to sense and adapt to the various acidic niches encountered during infection

Within the gastrointestinal tract, *K. pneumoniae* colonization has been linked to a putative RND-type efflux pump that enables increased acid tolerance and may be associated with the hypervirulent phenotype (26, 44). Enterohemorrhagic *E. coli* strains have likewise been shown to alter efflux activity and short-chain fatty acid composition of the outer membrane that provides cross-protection from antibiotics and the disinfectant triclosan (45, 46). Notably, in this study, growth in acidic pH alone did not alter overall permeability to novobiocin, which is used as an indicator for RND-type efflux pump activity. This indicates that other environmental signals than just pH may influence efflux pump expression and activity.

Our data indicate that the citrates and detergent components of Xperience alter membrane polarization and proton motive force (PMF). Alterations to PMF are strongly associated with bacterial tolerance responses when paired with active efflux systems (47). Furthermore, studies in *E. coli* have shown that rifampicin exposure induces a heterogeneous distribution of PMF activity within a clonal population, generating subpopulations of high and low PMF-active cells (48). Because PMF directly drives the uptake of certain antibiotic classes with intracellular targets, heterogeneity in PMF may contribute to the adaptive resistance observed in this study. The results presented here may therefore reflect this heterogeneous response, where individual bacteria alter membrane polarization in a non-coordinated manner, creating subpopulations with differential antibiotic uptake and susceptibility even within a genetically identical culture.

The data presented here suggest that exposure to an acidic pH can initiate a protective response without the involvement of either capsule or LPS O-antigen. This observation is supported by the recent large-scale investigation of how the permeability of the *Klebsiella* outer membrane influences susceptibility to multiple classes of antibiotics. Leus and Zgurskaya (9) found that permeability, and therefore susceptibility, to antibiotics was a fundamental property, independent of either porins or efflux pumps, and influenced by both the growth conditions of the bacteria and the concentration of the antibiotic. Studies in *E. coli* have further shown that antibiotic binding to charged outer membrane LPS and transit through the outer membrane is significantly impacted by both alterations to core oligosaccharide structure (49) and the ionic strength of the surrounding environment by altering lipid packing and phospholipid exposure to the outer environment (50). Together, these studies indicate that active remodeling of the outer membrane, including alterations to both porin expression and lipids, is a significant and distinctive mechanism for survival of *Klebsiella*. Our study is notable because we have linked this acid response with an adaptive resistance response that enhances active growth and survival in bacteriostatic concentrations of antibiotics.

Outer membrane remodeling in response to environmental stress is often facilitated by the production of outer membrane vesicles (OMVs) (51, 52). Vesicle production is known to increase in response to oxidative stress, and the rate of vesiculation and protein composition of OMVs have been shown to be influenced by the availability of iron (53). *Klebsiella* has also demonstrated the ability to survive within phagocytic cells, which requires managing the low pH of the phagolysosomal compartment and exposure to reactive oxygen species (54). The protein composition of *Klebsiella* OMVs has been shown to change with alterations to either LPS O-antigen or porin loss (55). OMVs from porin-loss isolates also exhibit a reduced host inflammatory response (56). OMV production to remodel membrane composition and permeability may be a key survival mechanism against multiple sources of cellular damage.

Porin loss in *Klebsiella* is a mechanism to enhance antibiotic resistance by limiting the transit of beta-lactams into the periplasmic space. The majority of porin loss isolates exhibit mutations in *ompK35* or *ompK36*, which eliminate expression of these transmembrane channels. In our studies, transcription of *ompK35* and *ompK36* was maintained, while expression of the alternative maltoporin *lamB* was dramatically decreased. These data concur with other studies that emphasize the critical role of *lamB* in beta-lactam resistance (37, 57–59). A recent survey of *Klebsiella* clinical isolates found that mutations in *lamB* significantly contribute to resistance to ceftazidime-avibactam combination therapy (60). *In vitro* studies have linked mutations in *lamB* with increased resistance to cefoxitin and meropenem but also revealed that *lamB* expression was required for *ompK36* porin loss (57). The *lamB* protein has also been shown to be downregulated in *E. coli* grown under acid conditions (61), which can now be connected with increased MICs to multiple classes of antibiotics.

Porin loss does not happen in isolation, as the downregulation of one porin is compensated for by upregulation of others (35). Enhanced expression of *ompA* and *lpp*, such as seen in this study*,* has been demonstrated in other studies in *Klebsiella* to enhance outer membrane stability, reduce permeability, and enhance resistance to antibiotics and virulence (36). These effects have been previously shown to enhance tolerance of exposure to reactive oxygen species or lysosomal enzymes (54), such as those found in an acidic phagolysosome. Porin loss mutations are also often paired with alterations in capsule and LPS expression (35) and can reduce the innate inflammatory response to *Klebsiella* (56). The data presented here indicate that responses to acidic pH do not require the capsule or LPS O-antigen to enhance bacterial tolerance of antibiotics, suggesting that low pH initiates distinct alterations in membrane permeability with limited effects on other known virulence factors.

Siderophores are established virulence factors of *K. pneumoniae* that scavenge iron from the host environment. *Klebsiella* produces multiple types of siderophores, each with distinct functional properties (10). The data presented here revealed that the production of salmochelin (*iroA*), the glucosylated form of enterobactin (*entB*), may be detrimental to the ability of the bacteria to survive antibiotics under acidic conditions. This finding is surprising, as other studies have shown deletion of siderophore genes to be detrimental to the overall survival and growth of both *Klebsiella* and *Salmonella* within host tissues (62, 63). Acidic pH is known to drive the reduction of iron bound by siderophores, enhancing the release of the iron molecule (64–66) and limiting the ability of siderophores to harvest iron from the environment. However, each siderophore also demonstrates a complex array of abilities to interact with and modulate host cells and tissues. Enterobactin has been shown to cross macrophage membranes, chelate intracellular iron, and skew macrophage polarization toward an M2, less microbicidal state (67, 68). *Klebsiella* clinical isolates with high siderophore expression exhibit limited oxidative stress during antibiotic exposure (69). *Klebsiella* has also been demonstrated to actively alter macrophage metabolic status to improve iron availability within a host cell (70), and siderophore expression directly impacts host inflammatory responses during pneumonia (63). Given these data, the adaptive resistance observed in *iroA* knockouts should be further investigated, as the lack of glucosylation of enterobactin in acidic environments may convey an advantage in colonization or intracellular survival.

The Xperience biofilm-disrupting lavage is formulated with a citric acid/sodium citrate buffering system that lowers pH and chelates divalent cations, such as iron. Treatment with Xperience aims to bind cations within bacterial extracellular matrices, and sodium lauryl sulfate acts as a surfactant to reduce surface tension and mechanically remove bacteria, while preserving host cell membrane integrity (12–14). As a citrate-buffered solution, Xperience has a substantially higher buffering capacity than LB. In contrast, adjusting LB pH to 4.5 with HCl produces minimal changes in ionic strength (calculated increase of ~ 8 mM), whereas the addition of diluted Xperience increases both ionic strength (~66.5 mM increase) and buffering capacity (~18 mM total citrate species) (71). This buffering difference is reflected in the level of pH stability observed after 24 h of *Klebsiella* growth. Dilute Xperience media showed more limited pH change, whereas pH 4.5 LB cultures exhibited greater pH drift. However, ionic strength does not appear to be the primary driver of the adaptive resistance response, as adaptive resistance was observed in both pH 4.5 LB and dilute Xperience media. This suggests that acidic pH itself, rather than the accompanying changes in ionic strength or buffering capacity, is the critical environmental signal triggering the adaptive resistance phenotype.

Xperience has been extensively tested on *S. aureus* and demonstrated efficacy in reducing surgical site infections in orthopedic surgery (72). Our data support this clinical finding, as Xperience was highly bacteriostatic to *S. aureus* even at very low dilutions and showed additive antibacterial effects on *E. coli*. The enhanced bacteriostatic effect in *E. coli* directly demonstrates that these dilute solutions are not hydrolyzing beta-lactam antibiotics, as can occur at acidic pH (73), but rather triggering species-specific physiological responses. Colistin experiments further support this conclusion, as colistin is stabilized in acidic solutions and should maintain or enhance membrane destabilization activity (74); however, adaptive resistance still occurred in *Klebsiella*.

These findings emphasize that each bacterial species has distinct adaptive capacities to general antimicrobial treatments. While Xperience maintains bacteriostatic activity against *Klebsiella* at full strength, dilution of the lavage fluid through tissue adsorption *in vivo* could create subinhibitory concentrations that initiate the adaptive resistance response. This concern extends beyond surgical lavage applications as citrate buffers are commonly used in injectable drug formulations (75), and dilution of these buffering agents into the bloodstream could create localized acidic microenvironments that trigger the adaptive resistance phenotype in colonizing *Klebsiella*.

The data presented here highlight the fact that antibiotic resistance comes in multiple forms. While genotypic resistance via mutation or acquisition of specific genes is well known, the initial adaptive response that leads to these genetically altered isolates likely involves radical changes in gene expression to allow bacterial cells time to acquire new genes or mutate their way to survival. Adaptive resistance responses can lead to quiescent persister cells that survive without growing in the presence of an antibiotic or tolerant cells, which slow the rate of killing by an antibiotic but do not alter the overall MIC (5, 6, 76). The active growth and significantly elevated MIC observed in this study indicate that *Klebsiella* is responding with a distinct response that is neither persistence nor true tolerance and illustrate the power of changes in gene expression to facilitate resistance. Our observations are supported by a recent study by Beagle and Levin that detailed how upregulation of penicillin-binding proteins in acid-treated *Klebsiella* elevates MICs (77), further emphasizing the importance of the initial transcriptional response to antibiotic exposure.

The transcriptional changes triggered by acidic pH may be an initial step in the evolution of a resistant *Klebsiella* isolate and the acquisition of traditional resistance genes. Citric acid treatment is known to improve transformation efficiency in hypervirulent *Klebsiella* (78) and to induce plasmid-mediated cefiderocol resistance via a ferric citrate transport system (79). Given this, these data strongly suggest that the response to acid pH and citrate activates a suite of changes involving iron binding and transport, as well as the uptake of external DNA that can facilitate the acquisition and expression of newly acquired resistance genes.

In summary, this study demonstrates how an acidic environment can alter gene expression to allow *Klebsiella* to activate a nonspecific mechanism to survive multiple classes of antibiotics. These data add to studies that demonstrate the ability of *Klebsiella* to achieve genotypic clinical resistance without the traditional acquisition of new beta-lactamase genes after sub-MIC antibiotic exposure (80). Antibiotic resistance studies have traditionally focused on drug concentration, or selection window, as the main parameter for emerging resistant isolates (81, 82). The data presented here expand on this idea to encompass the physiological environment of the bacteria during that drug exposure. Further investigation is needed to characterize the many environmental conditions other than antibiotic concentration that can open the selection window for the emergence of tolerant, persister, or fully resistant bacterial cells.

## Data Availability

Complete genome sequences are available via NCBI BioProject database. The project ID is PRJNA1468276. All other data are available upon request.

## SUPPLEMENTAL MATERIAL

Supplemental tables and figure spectrum.00243-26-s0001.pdf Figure S1; Tables S1 and S2.

ASM does not own the copyrights to Supplemental Material that may be linked to, or accessed through, an article. The authors have granted ASM a non-exclusive, world-wide license to publish the Supplemental Material files. Please contact the corresponding author directly for reuse.

## References

[1] Paczosa MK, Mecsas J. 2016. *Klebsiella pneumoniae*: going on the offense with a strong defense. Microbiol Mol Biol Rev 80:629–661. doi:10.1128/MMBR.00078-15 27307579 PMC4981674

[2] Pendleton JN, Gorman SP, Gilmore BF. 2013. Clinical relevance of the ESKAPE pathogens. Expert Rev Anti Infect Ther 11:297–308. doi:10.1586/eri.13.12 23458769

[3] Li J, Shi Y, Song X, Yin X, Liu H. 2025. Mechanisms of antimicrobial resistance in *Klebsiella*: advances in detection methods and clinical implications. Infect Drug Resist 18:1339–1354. doi:10.2147/IDR.S509016 40092844 PMC11910031

[4] Nikaido H. 2003. Molecular basis of bacterial outer membrane permeability revisited. Microbiol Mol Biol Rev 67:593–656. doi:10.1128/MMBR.67.4.593-656.2003 14665678 PMC309051

[5] Niu H, Gu J, Zhang Y. 2024. Bacterial persisters: molecular mechanisms and therapeutic development. Signal Transduct Target Ther 9:174. doi:10.1038/s41392-024-01866-5 39013893 PMC11252167

[6] Balaban NQ, Helaine S, Lewis K, Ackermann M, Aldridge B, Andersson DI, Brynildsen MP, Bumann D, Camilli A, Collins JJ, Dehio C, Fortune S, Ghigo J-M, Hardt W-D, Harms A, Heinemann M, Hung DT, Jenal U, Levin BR, Michiels J, Storz G, Tan M-W, Tenson T, Van Melderen L, Zinkernagel A. 2019. Definitions and guidelines for research on antibiotic persistence. Nat Rev Microbiol 17:441–448. doi:10.1038/s41579-019-0196-3 30980069 PMC7136161

[7] Martínez-Martínez L. 2008. Extended-spectrum beta-lactamases and the permeability barrier. Clin Microbiol Infect 14 Suppl 1:82–89. doi:10.1111/j.1469-0691.2007.01860.x 18154531

[8] Rocker A, Lacey JA, Belousoff MJ, Wilksch JJ, Strugnell RA, Davies MR, Lithgow T. 2020. Global trends in proteome remodeling of the outer membrane modulate antimicrobial permeability in *Klebsiella pneumoniae*. mBio 11:e00603-20. doi:10.1128/mBio.00603-20 32291303 PMC7157821

[9] Leus IV, Zgurskaya HI. 2025. No two are alike: on the role of *Klebsiella pneumoniae* permeability barriers in antibiotic susceptibility and persistence. Antimicrob Agents Chemother 69:e0008525. doi:10.1128/aac.00085-25 40525411 PMC12327008

[10] Kumar A, Chakravorty S, Yang T, Russo TA, Newton SM, Klebba PE. 2024. Siderophore-mediated iron acquisition by *Klebsiella pneumoniae* J Bacteriol 206:e0002424. doi:10.1128/jb.00024-24 38591913 PMC11112993

[11] Holt KE, Wertheim H, Zadoks RN, Baker S, Whitehouse CA, Dance D, Jenney A, Connor TR, Hsu LY, Severin J, et al.. 2015. Genomic analysis of diversity, population structure, virulence, and antimicrobial resistance in *Klebsiella pneumoniae*, an urgent threat to public health. Proc Natl Acad Sci USA 112. doi:10.1073/pnas.1501049112 PMC4500264 26100894

[12] Gaur G, Predtechenskaya M, Voyich JM, James G, Stewart PS, Borgogna TR. 2024. Assessing the effects of surgical irrigation solutions on human neutrophil interactions with nascent *Staphylococcus aureus* biofilms. Microorganisms 12:1951. doi:10.3390/microorganisms12101951 39458262 PMC11509154

[13] Vatti L, Gopinath R, Heshmat C, Lariosa S, Rabbitt S, Bashyal R. 2024. The use of a novel surgical irrigant may be associated with decreased incidence of surgical site infections. Journal of Orthopaedic Experience & Innovation 5. doi:10.60118/001c.121295

[14] Ng MK, Emara A, Salman M, Mastrokostas PG, Razi AE. 2025. Novel citrate-based wound irrigation system disrupting biofilms and preventing orthopaedic surgery infections: technique guide and systematic review. J Spine Surg 11:678–687. doi:10.21037/jss-25-45 41089909 PMC12516406

[15] Shankar-Sinha S, Valencia GA, Janes BK, Rosenberg JK, Whitfield C, Bender RA, Standiford TJ, Younger JG. 2004. The *Klebsiella pneumoniae* o antigen contributes to bacteremia and lethality during murine pneumonia. Infect Immun 72:1423–1430. doi:10.1128/IAI.72.3.1423-1430.2004 14977947 PMC355988

[16] Walker KA, Treat LP, Sepúlveda VE, Miller VL. 2020. The small protein rmpd drives hypermucoviscosity in *Klebsiella pneumoniae*. mBio 11:1–14. doi:10.1128/mBio.01750-20 PMC7512549 32963003

[17] Bachman MA, Lenio S, Schmidt L, Oyler JE, Weiser JN. 2012. Interaction of lipocalin 2, transferrin, and siderophores determines the replicative niche of *Klebsiella pneumoniae* during pneumonia. mBio 3:e00224-11. doi:10.1128/mBio.00224-11 23169997 PMC3509427

[18] Kadeřábková N, Mahmood AJS, Mavridou DAI. 2024. Antibiotic susceptibility testing using minimum inhibitory concentration (MIC) assays. NPJ Antimicrob Resist 2:37. doi:10.1038/s44259-024-00051-6 39843555 PMC11721449

[19] Llobet E, March C, Giménez P, Bengoechea JA. 2009. *Klebsiella pneumoniae* OmpA confers resistance to antimicrobial peptides. Antimicrob Agents Chemother 53:298–302. doi:10.1128/AAC.00657-08 19015361 PMC2612193

[20] Buttress JA, Halte M, Te Winkel JD, Erhardt M, Popp PF, Strahl H. 2022. A guide for membrane potential measurements in gram-negative bacteria using voltage-sensitive dyes. Microbiology (Reading) 168. doi:10.1099/mic.0.001227 36165741

[21] Bellio P, Fagnani L, Nazzicone L, Celenza G. 2021. New and simplified method for drug combination studies by checkerboard assay. MethodsX 8:101543. doi:10.1016/j.mex.2021.101543 34754811 PMC8563647

[22] Budnick JA, Bina XR, Bina JE. 2021. Complete genome sequence of *Klebsiella pneumoniae* strain ATCC 43816. Microbiol Resour Announc 10:e01441-20. doi:10.1128/MRA.01441-20 33541885 PMC7862963

[23] Plasmidsaurus. 2026. Whole Genome Technical Documentation. Available from: https://plasmidsaurus.com/technical-documentation/genome

[24] Schmittgen TD, Livak KJ. 2001. Analysis of relative gene expression data using real-time quantitative PCR and the 2(-Delta Delta C(T)) Method. Methods 25:402–408. doi:10.1006/meth.2001.1262 11846609

[25] Real Statistics Resource Pack. Real Statistics Using Excel. Available from: https://real-statistics.com/free-download/real-statistics-resource-pack. Retrieved 1414 MayMay 2026. Accessed , 1414 MayMay 2026

[26] Van Allen ME, Weng Y, Bina XR, Bina JE. 2025. Inhibition of RND-mediated efflux attenuates antibiotic resistance and virulence in hypervirulent *Klebsiella pneumoniae.* Infect Immun 93:e0030125. doi:10.1128/iai.00301-25 40980928 PMC12519776

[27] Wijers CDM, Pham L, Douglass MV, Skaar EP, Palmer LD, Noto MJ. 2022. Gram-negative bacteria act as a reservoir for aminoglycoside antibiotics that interact with host factors to enhance bacterial killing in a mouse model of pneumonia. FEMS Microbes 3:xtac016. doi:10.1093/femsmc/xtac016 35909464 PMC9326624

[28] Lang M, Carvalho A, Baharoglu Z, Mazel D. 2023. Aminoglycoside uptake, stress, and potentiation in gram-negative bacteria: new therapies with old molecules. Microbiol Mol Biol Rev 87:e0003622. doi:10.1128/mmbr.00036-22 38047635 PMC10732077

[29] Holden VI, Bachman MA. 2015. Diverging roles of bacterial siderophores during infection. Metallomics 7:986–995. doi:10.1039/c4mt00333k 25745886

[30] Gootz TD. 2010. The global problem of antibiotic resistance. Crit Rev Immunol 30:79–93. doi:10.1615/critrevimmunol.v30.i1.60 20370622

[31] Siu LK. 2001. Is OmpK35 specific for ceftazadime penetration? Antimicrob Agents Chemother 45:1601–1602. doi:10.1128/AAC.45.5.1601-1602.2001 11372640 PMC90518

[32] Doménech-Sánchez A, Martínez-Martínez L, Hernández-Allés S, del Carmen Conejo M, Pascual A, Tomás JM, Albertí S, Benedí VJ. 2003. Role of *Klebsiella pneumoniae* OmpK35 porin in antimicrobial resistance. Antimicrob Agents Chemother 47:3332–3335. doi:10.1128/AAC.47.10.3332-3335.2003 14506051 PMC201126

[33] Tsai Y-K, Fung C-P, Lin J-C, Chen J-H, Chang F-Y, Chen T-L, Siu LK. 2011. *Klebsiella pneumoniae* outer membrane porins OmpK35 and OmpK36 play roles in both antimicrobial resistance and virulence. Antimicrob Agents Chemother 55:1485–1493. doi:10.1128/AAC.01275-10 21282452 PMC3067157

[34] Shakib P, Ghafourian S, Zolfaghary MR, Hushmandfar R, Ranjbar R, Sadeghifard N. 2012. Prevalence of OmpK35 and OmpK36 porin expression in beta-lactamase and non-betalactamase- producing *Klebsiella pneumoniae*. Biologics 6:1–4. doi:10.2147/BTT.S27582 22291461 PMC3266860

[35] Brunson DN, Maldosevic E, Velez A, Figgins E, Ellis TN. 2019. Porin loss in *Klebsiella pneumoniae* clinical isolates impacts production of virulence factors and survival within macrophages. Int J Med Microbiol 309:213–224. doi:10.1016/j.ijmm.2019.04.001 31010630

[36] March C, Cano V, Moranta D, Llobet E, Pérez-Gutiérrez C, Tomás JM, Suárez T, Garmendia J, Bengoechea JA. 2013. Role of bacterial surface structures on the interaction of *Klebsiella pneumoniae* with phagocytes. PLoS One 8:e56847. doi:10.1371/journal.pone.0056847 23457627 PMC3574025

[37] García-Sureda L, Juan C, Doménech-Sánchez A, Albertí S. 2011. Role of *Klebsiella pneumoniae* LamB porin in antimicrobial resistance. Antimicrob Agents Chemother 55:1803–1805. doi:10.1128/AAC.01441-10 21282437 PMC3067167

[38] Diedrich DL, Fralick JA. 1982. Relationship between the OmpC and LamB proteins of *Escherichia coli* and its influence on the protein mass of the outer membrane. J Bacteriol 149:156–160. doi:10.1128/jb.149.1.156-160.1982 7033205 PMC216604

[39] Abhinand K, Menon AM, Thomas SS, Anil AB, Parvathi Mohanan PC, Arun KB, Edison LK, Babu P, Kumar GB, Nair BG, Madhavan A. 2025. *Klebsiella pneumoniae*: Host interactions, virulence mechanisms, and novel therapeutic strategies. Microb Pathog 207:107856. doi:10.1016/j.micpath.2025.107856 40602443

[40] Marunaka Y. 2015. Roles of interstitial fluid pH in diabetes mellitus: glycolysis and mitochondrial function. World J Diabetes 6:125–135. doi:10.4239/wjd.v6.i1.125 25685283 PMC4317304

[41] Stiefel U, Rao A, Pultz MJ, Jump RLP, Aron DC, Donskey CJ. 2006. Suppression of gastric acid production by proton pump inhibitor treatment facilitates colonization of the large intestine by vancomycin-resistant *Enterococcus spp*. and *Klebsiella pneumoniae* in clindamycin-treated mice. Antimicrob Agents Chemother 50:3905–3907. doi:10.1128/AAC.00522-06 16940078 PMC1635221

[42] Koskinen K, Penttinen R, Örmälä-Odegrip AM, Giske CG, Ketola T, Jalasvuori M. 2021. Systematic comparison of epidemic and non-epidemic carbapenem resistant *Klebsiella pneumoniae* strains. Front Cell Infect Microbiol 11:599924. doi:10.3389/fcimb.2021.599924 33708644 PMC7940544

[43] Xu Y, Zhao Z, Tong W, Ding Y, Liu B, Shi Y, Wang J, Sun S, Liu M, Wang Y, Qi Q, Xian M, Zhao G. 2020. An acid-tolerance response system protecting exponentially growing *Escherichia coli*. Nat Commun 11. doi:10.1038/s41467-020-15350-5 PMC7083825 32198415

[44] Coudeyras S, Nakusi L, Charbonnel N, Forestier C. 2008. A tripartite efflux pump involved in gastrointestinal colonization by *Klebsiella pneumoniae* confers a tolerance response to inorganic acid. Infect Immun 76:4633–4641. doi:10.1128/IAI.00356-08 18644883 PMC2546844

[45] Sheikh SW, Ali A, Ahsan A, Shakoor S, Shang F, Xue T. 2021. Insights into emergence of antibiotic resistance in acid-adapted enterohaemorrhagic escherichia coli. Antibiotics (Basel) 10:522. doi:10.3390/antibiotics10050522 34063307 PMC8147483

[46] Karatzas KAG, Webber MA, Jorgensen F, Woodward MJ, Piddock LJV, Humphrey TJ. 2007. Prolonged treatment of *Salmonella enterica* serovar typhimurium with commercial disinfectants selects for multiple antibiotic resistance, increased efflux and reduced invasiveness. J Antimicrob Chemother 60:947–955. doi:10.1093/jac/dkm314 17855722

[47] Wan Y, Zheng J, Chan EWC, Chen S. 2024. Proton motive force and antibiotic tolerance in bacteria. Microb Biotechnol 17:e70042. doi:10.1111/1751-7915.70042 39487809 PMC11531170

[48] Lee AH, Gupta R, Nguyen HN, Schmitz IR, Siegele DA, Lele PP. 2023. Heterogeneous distribution of proton motive force in nonheritable antibiotic resistance. mBio 14:e0238422. doi:10.1128/mbio.02384-22 36598258 PMC9973297

[49] Clifton LA, Ciesielski F, Skoda MWA, Paracini N, Holt SA, Lakey JH. 2016. The effect of lipopolysaccharide core oligosaccharide size on the electrostatic binding of antimicrobial proteins to models of the gram negative bacterial outer membrane. Langmuir 32:3485–3494. doi:10.1021/acs.langmuir.6b00240 27003358 PMC4854487

[50] Snyder DS, McIntosh TJ. 2000. The lipopolysaccharide barrier: correlation of antibiotic susceptibility with antibiotic permeability and fluorescent probe binding kinetics. Biochemistry 39:11777–11787. doi:10.1021/bi000810n 10995246

[51] McBroom AJ, Johnson AP, Vemulapalli S, Kuehn MJ. 2006. Outer membrane vesicle production by *Escherichia coli* is independent of membrane instability. J Bacteriol 188:5385–5392. doi:10.1128/JB.00498-06 16855227 PMC1540050

[52] Mozaheb N, Mingeot-Leclercq MP. 2020. Membrane vesicle production as a bacterial defense against stress. Front Microbiol 11:600221. doi:10.3389/fmicb.2020.600221 33362747 PMC7755613

[53] Płaczkiewicz J, Gieczewska K, Musiałowski M, Adamczyk-Popławska M, Bącal P, Kwiatek A. 2023. Availability of iron ions impacts physicochemical properties and proteome of outer membrane vesicles released by *Neisseria gonorrhoeae*. Sci Rep 13:18733. doi:10.1038/s41598-023-45498-1 37907530 PMC10618220

[54] Cano V, March C, Insua JL, Aguiló N, Llobet E, Moranta D, Regueiro V, Brennan GP, Millán-Lou MI, Martín C, Garmendia J, Bengoechea JA. 2015*.* *Klebsiella pneumoniae* survives within macrophages by avoiding delivery to lysosomes. Cell Microbiol 17:1537–1560. doi:10.1111/cmi.12466 26045209

[55] Cahill BK, Seeley KW, Gutel D, Ellis TN. 2015. *Klebsiella pneumoniae* o antigen loss alters the outer membrane protein composition and the selective packaging of proteins into secreted outer membrane vesicles. Microbiol Res 180:1–10. doi:10.1016/j.micres.2015.06.012 26505306

[56] Turner KL, Cahill BK, Dilello SK, Gutel D, Brunson DN, Albertí S, Ellis TN. 2015. Porin loss impacts the host inflammatory response to outer membrane vesicles of *Klebsiella pneumoniae**.* Antimicrob Agents Chemother 60:1360–1369. doi:10.1128/AAC.01627-15 26666932 PMC4776010

[57] García-Sureda L, Doménech-Sánchez A, Barbier M, Juan C, Gascó J, Albertí S. 2011. OmpK26, a novel porin associated with carbapenem resistance in *Klebsiella pneumoniae*. Antimicrob Agents Chemother 55:4742–4747. doi:10.1128/AAC.00309-11 21807980 PMC3186958

[58] Lin X, Yang M, Li H, Wang C, Peng X-X. 2014. Decreased expression of LamB and Odp1 complex is crucial for antibiotic resistance in *Escherichia coli*. J Proteomics 98:244–253. doi:10.1016/j.jprot.2013.12.024 24412198

[59] Li W, Wang G, Zhang S, Fu Y, Jiang Y, Yang X, Lin X. 2019. An integrated quantitative proteomic and metabolomics approach to reveal the negative regulation mechanism of LamB in antibiotics resistance. J Proteomics 194:148–159. doi:10.1016/j.jprot.2018.11.022 30521976

[60] Guo Y, Liu N, Lin Z, Ba X, Zhuo C, Li F, Wang J, Li Y, Yao L, Liu B, Xiao S, Jiang Y, Zhuo C. 2021. Mutations in porin LamB contribute to ceftazidime-avibactam resistance in KPC-producing *Klebsiella pneumoniae* Emerg Microbes Infect 10:2042–2051. doi:10.1080/22221751.2021.1984182 34551677 PMC8567916

[61] Wu L, Lin X, Peng X. 2009. From proteome to genome for functional characterization of pH-dependent outer membrane proteins in *Escherichia coli*. J Proteome Res 8:1059–1070. doi:10.1021/pr800818r 19132928

[62] Nagy TA, Moreland SM, Andrews-Polymenis H, Detweiler CS. 2013. The ferric enterobactin transporter fep is required for persistent *Salmonella enterica* serovar typhimurium infection. Infect Immun 81:4063–4070. doi:10.1128/IAI.00412-13 23959718 PMC3811836

[63] Holden VI, Breen P, Houle S, Dozois CM, Bachman MA. 2016. *Klebsiella pneumoniae* siderophores induce inflammation, bacterial dissemination, and HIF-1ɑ stabilization during pneumonia. mBio 7. doi:10.1128/mBio.01397-16 PMC5021805 27624128

[64] Dhungana S, Crumbliss AL. 2005. Coordination chemistry and redox processes in siderophore-mediated iron transport. Geomicrobiol J 22:87–98. doi:10.1080/01490450590945870

[65] Miethke M, Marahiel MA. 2007. Siderophore-based iron acquisition and pathogen control. Microbiol Mol Biol Rev 71:413–451. doi:10.1128/MMBR.00012-07 17804665 PMC2168645

[66] Abergel RJ, Warner JA, Shuh DK, Raymond KN. 2006. Enterobactin protonation and iron release: structural characterization of the salicylate coordination shift in ferric enterobactin. J Am Chem Soc 128:8920–8931. doi:10.1021/ja062046j 16819888 PMC3188320

[67] Saha P, Xiao X, Yeoh BS, Chen Q, Katkere B, Kirimanjeswara GS, Vijay-Kumar M. 2019. The bacterial siderophore enterobactin confers survival advantage to *Salmonella* in macrophages. Gut Microbes 10:412–423. doi:10.1080/19490976.2018.1546519 30449241 PMC6546333

[68] Saha P, Xiao X, Yeoh BS, Chen Q, Katkere B, Kirimanjeswara GS, Vijay-Kumar M. 2018. Dual roles of the bacterial siderophore enterobactin by inducing apoptosis in macrophages and promoting survival advantages to *Salmonella typhimurium*. The Journal of Immunology 200:114. doi:10.4049/jimmunol.200.Supp.114.3

[69] Zhang W, Zhang Y, Wang X, Ding F, Fu Y, Zhao J, Song W, Opiyo OJ, Zhang F, Chen X. 2017. Siderophores in clinical isolates of *Klebsiella pneumoniae* promote ciprofloxacin resistance by inhibiting the oxidative stress. Biochem Biophys Res Commun 491:855–861. doi:10.1016/j.bbrc.2017.04.108 28435070

[70] Grubwieser P, Hilbe R, Gehrer CM, Grander M, Brigo N, Hoffmann A, Seifert M, Berger S, Theurl I, Nairz M, Weiss G. 2023. *Klebsiella pneumoniae* manipulates human macrophages to acquire iron. Front Microbiol 14:1223113. doi:10.3389/fmicb.2023.1223113 37637102 PMC10451090

[71] Calculating Ionic strength of buffers – the hancock lab. 2026. Calculating ionic strength of buffers – the hancock lab. Available from: https://sites.psu.edu/hancocklab/2024/03/29/calculating-ionic-strength-of-buffers. Retrieved 21 May 2026.

[72] Williams M, Harris RM. 2024. Efficacy of a novel intraoperative surgical irrigant in preventing periprosthetic joint infections in primary knee, hip, and shoulder arthroplasties: a retrospective analysis. Orthop Surg 16:1277–1283. doi:10.1111/os.14052 38627352 PMC11144508

[73] Mitchell SM, Ullman JL, Teel AL, Watts RJ. 2014. PH and temperature effects on the hydrolysis of three β-lactam antibiotics: ampicillin, cefalotin and cefoxitin. Sci Total Environ 466–467:547–555. doi:10.1016/j.scitotenv.2013.06.027 23948499

[74] Orwa JA, Govaerts C, Gevers K, Roets E, Van Schepdael A, Hoogmartens J. 2002. Study of the stability of polymyxins B(1), E(1) and E(2) in aqueous solution using liquid chromatography and mass spectrometry. J Pharm Biomed Anal 29:203–212. doi:10.1016/s0731-7085(02)00016-x 12062679

[75] Lambros M, Tran TH, Fei Q, Nicolaou M. 2022. Citric acid: a multifunctional pharmaceutical excipient. Pharmaceutics 14:972. doi:10.3390/pharmaceutics14050972 35631557 PMC9148065

[76] Zhou Y, Liao H, Pei L, Pu Y. 2023. Combatting persister cells: the daunting task in post-antibiotics era. Cell Insight 2:100104. doi:10.1016/j.cellin.2023.100104 37304393 PMC10250163

[77] Beagle S, Levin PA. 2026. Acid-dependent beta-lactam resistance in *Klebsiella pneumoniae* is mediated by paralogous class B PBPs and the class A PBP, PBP1b. mBio 17:e0009226. doi:10.1128/mbio.00092-26 42112825 PMC13251458

[78] Park S, Ko KS. 2023. Improvement of transformation efficiency in hypermucoviscous *Klebsiella pneumoniae* using citric acid. J Microbiol Methods 205:106673. doi:10.1016/j.mimet.2023.106673 36638870

[79] Polani R, De Francesco A, Tomolillo D, Artuso I, Equestre M, Trirocco R, Arcari G, Antonelli G, Villa L, Prosseda G, Visca P, Carattoli A. 2025. Cefiderocol resistance conferred by plasmid-located ferric citrate transport system in KPC-producing *Klebsiella pneumoniae*. Emerg Infect Dis 31:123–124. doi:10.3201/eid3101.241426 39714320 PMC11682805

[80] Anderson JR, Lam NB, Jackson JL, Dorenkott SM, Ticer T, Maldosevic E, Velez A, Camden MR, Ellis TN. 2023. Progressive sub-MIC exposure of *Klebsiella pneumoniae* 43816 to cephalothin induces the evolution of beta-lactam resistance without acquisition of beta-lactamase genes. Antibiotics (Basel) 12:887. doi:10.3390/antibiotics12050887 37237790 PMC10215138

[81] Drlica K. 2003. The mutant selection window and antimicrobial resistance. J Antimicrob Chemother 52:11–17. doi:10.1093/jac/dkg269 12805267

[82] Drlica K, Zhao X. 2007. Mutant selection window hypothesis updated. Clin Infect Dis 44:681–688. doi:10.1086/511642 17278059

