# Supplementary material for: Acid exposure induces adaptive resistance in Klebsiella pneumoniae to combination treatment with antibiotics and biofilm-disrupting surgical lavage
Source: Microbiol Spectr. 2026 Jul 16;14(8):e00243-26. doi: 10.1128/spectrum.00243-26 (PMC13435878; doi:10.1128/spectrum.00243-26)
Supplement: Supplementary file 1 [file spectrum.00243-26-s0001.pdf]

Supplemental Data for Chirumbolo et al. :

Acid Exposure Induces Adaptive Resistance in *Klebsiella pneumoniae* to Combination Treatment with Antibiotics and Biofilm Disrupting Surgical Lavage

Supplemental Table 1. Primers used for qPCR.

	**Primer Sequence (5' -→ 3')**	
**Bacterial Gene**	**Forward primer**	**Reverse primer**
gapA	TTGACCTGACCGTTCGTCTGGAAA	AGCATCGAACACGGAAGTGCAAAC
ompK35	TTCGACAACGCTATCGCACTGTCT	AGTACATGACGGCCGCATAGATGT
ompK36	CCGTCAACCAGACCGAAGAA	CAGGCCTGAAATTTGGCGAC
ompK26	GAACAACGCCCGGCAAGATGATGA	AGCTGCGGGCATAGACATAGTTCA
lamB	GCGGGTAAACGCTTCTATCA	GGTCAACGTTTTCCAGACCT
ompA	ACGTGCTCAGTCCGTTGTTGACTA	AGTAACCGGGTTGGATTCACCCAT
lpp	CGGTAATCCTGGGTTCTACTCT	TGCTCAGCTGGTCAACTTTAG
phoQ	GTAACTTGCGTCTGGTGGTGAA	CGTTCCGGGTCAAACTTCTCAA
rpoS	ATGAACCAAACCCGTACGATCC	GGTCCAGCTTATGGGACAACTC
soxS	ACTCAGCGGCCGATCTTTGATA	GCGGAGATCTGATGGCGATAGT

Supplemental Figure 1: Checkerboard Assay Results using *Staphylococcus aureus* 6538

S. aureus 6538	Cephalotl	none	1 ug/ml	2 ug/ml	4 ug/ml	8 ug/ml	16 ug/ml	32 ug/ml	64 ug/ml	125 ug/ml	250 ug/ml	500 ug.ml	1000 ug/m	XP
	A	0.044	0.044	0.043	0.043	0.044	0.044	0.044	0.043	0.042	0.042	0.042	0.048	1/2
	B	0.056	0.057	0.055	0.058	0.058	0.055	0.055	0.054	0.052	0.051	0.048	0.048	1/4
	C	0.075	0.07	0.069	0.07	0.073	0.07	0.07	0.072	0.073	0.073	0.073	0.071	1/8
	D	0.065	0.065	0.066	0.064	0.063	0.065	0.066	0.065	0.071	0.071	0.077	0.073	1/16
	E	0.052	0.053	0.053	0.052	0.052	0.075	0.053	0.055	0.056	0.057	0.061	0.059	1/32
	F	0.046	0.045	0.045	0.045	0.045	0.047	0.046	0.046	0.046	0.046	0.048	0.048	1/64
	G	0.046	0.045	0.044	0.045	0.045	0.046	0.045	0.044	0.044	0.045	0.046	0.045	1/128
	H	0.399	0.044	0.045	0.05	0.046	0.045	0.046	0.046	0.044	0.044	0.045	0.043	none

S. aureus 6538	Cephalotl	none	1 ug/ml	2 ug/ml	4 ug/ml	8 ug/ml	16 ug/ml	32 ug/ml	64 ug/ml	125 ug/ml	250 ug/ml	500 ug.ml	1000 ug/m	XP
	A	0.047	0.044	0.043	0.043	0.043	0.044	0.044	0.044	0.042	0.042	0.042	0.044	**1/2**
	B	0.059	0.071	0.058	0.057	0.055	0.062	0.058	0.056	0.054	0.053	0.048	0.048	**1/4**
	C	0.076	0.081	0.076	0.077	0.078	0.074	0.08	0.079	0.08	0.078	0.083	0.077	**1/8**
	D	0.07	0.069	0.069	0.072	0.074	0.07	0.094	0.077	0.073	0.079	0.085	0.084	**1/16**
	E	0.056	0.055	0.054	0.055	0.055	0.056	0.055	0.056	0.057	0.06	0.068	0.065	**1/32**
	F	0.047	0.046	0.046	0.046	0.046	0.048	0.046	0.046	0.046	0.047	0.05	0.049	**1/64**
	G	0.045	0.045	0.045	0.045	0.046	0.046	0.045	0.045	0.045	0.045	0.046	0.046	**1/128**
	H	0.705	0.044	0.045	0.044	0.046	0.046	0.046	0.046	0.045	0.044	0.044	0.044	**none**

Figure 1- Raw OD reads of *S. aureus* checkerboard assay with Xperience and Cephalothin. The untreated growth control (bacteria + broth only) is found in well H8 of each plate. No growth was observed when treated with either Xpereince or Cephalothin alone or in combination, at all doses and dilutions tested.

Supplemental Table 2: : Whole Genome Sequencing SNPs from 1 or 5 day exposure. All variants determined compared to WGS sequence of *K. pneumoniae* grown in parallel in neutral pH LB, no antibiotics added.

**Treatment**	**All treatments**	**All treatments**	**LB pH 4.5 + Cephalothin**	**LB pH 4.5 + Cephalothin**	**LB pH 4.5 + Cephalothin**	**LB pH 4.5 + Cephalothin**	**LB pH 4.5 + Cephalothin**	**LB pH 4.5 + Cephalothin**	**1:16 XP**
**Exposure (Days)**	1	5	1	1	1	1	1	5	5
**CHROM**	CP064352.1	CP064352.1	CP064352.1	CP064352.1	CP064352.1	CP064352.1	CP064352.1	CP064352.1	CP064352.1
**Position**	5215	5215	685327	685387	331463	685150	5351406	330839	3505340
**Coding Sequence**			39/2343		541/1098	216/2343	384/510		
**REF**	G	G	A	C	G	C	G	A	CA
**ALT**	GGA	GGA	C	CG	T	G	T	G	C
**Gene**	IT767_00025	IT767_00025	IT767_03120	IT767_03120	IT767_01530	IT767_03120	IT767_25745	*rcsB*	IT767_16780
**Gene ID**	IT767_00025	IT767_00025	IT767_03120	IT767_03120	IT767_01530	IT767_03120	IT767_25745	IT767_01520	IT767_16780
**Consequence**	Upstream gene variant	Upstream gene variant	Synonymous variant	Upstream gene variant	Stop gained	Synonymous variant	Missense variant	Upstream gene variant	Upstream gene variant
**Impact**	MODIFIER	MODIFIER	LOW	MODIFIER	HIGH	LOW	MODERATE	MODIFIER	MODIFIER
